# Overlapping Hyaline Fibromatosis Syndrome: A Rare Case of Juvenile Hyaline Fibromatosis and Infantile Systemic Hyalinosis

**DOI:** 10.7759/cureus.27947

**Published:** 2022-08-12

**Authors:** Rupal M Oswal, Shruthi S Prasad, Naveen Manohar, Gajanan A Pise, Kiran Rao

**Affiliations:** 1 Dermatology, Belagavi Institute of Medical Sciences, Belagavi, IND; 2 Dermatology, Kasturba Medical College, Manipal, Manipal, IND

**Keywords:** histopathology examination, allelic autosomal recessive disorders, juvenile hyaline fibromatosis, infantile systemic hyalinosis, hyaline fibromatosis syndrome

## Abstract

Juvenile hyaline fibromatosis (JHF) and infantile systemic hyalinosis (ISF) are rare progressive, fatal autosomal recessive fibromatosis disorders that are characterized by the deposition of hyaline in various tissues. Mutations in capillary morphogenesis gene 2 are responsible for both of these conditions. These disorders usually present with fleshy, papular lesions, joint contractures, gingival hyperplasia, and persistent diarrhoea. An 18-month-old boy presented with multiple scalp abscesses, facial nodules, gingival hypertrophy, hypertrophic verrucous plaques and joint contractures with unique dermoscopic features and a history of recurrent diarrhoea and infections. Histopathological examination following skin biopsy revealed deposition of hyaline in the stroma and subcutaneous tissues. JHF is a differential diagnosis in children who present with multiple scalp nodules. Here, we report the case of overlapping features of JHF and ISH. The evolution of this case provides a special opportunity to further understand the pathogenesis and clinical characterization of hyaline fibromatosis syndrome.

## Introduction

Juvenile hyaline fibromatosis (JHF) and infantile systemic hyalinosis (ISH) are rare allelic autosomal recessive disorders due to 4q21.21 mutations [1] that present with papulonodular cutaneous lesions, joint contractures, gingival hypertrophy, osteoporosis, recurrent infections, persistent diarrhoea, and visceral involvement. Cutaneous lesions in JHF include nodules and/or papules over the head and neck; other lesions include large tumours, especially, on the scalp, trunk, and limbs and perianal plaques or nodules. In contrast, ISH includes articular contractures, gingival hypertrophy, bone abnormalities, and thickened skin with hyperpigmentation over bony prominences. The hallmark of these diseases is the deposition of amorphous hyaline material in various tissues. The amorphous hyaline material is principally composed of glycoproteins and glycosaminoglycans, and its origin remains unclear [1]. Herein, we report a rare case of overlapping features of JHF and ISH.

## Case presentation

**Figure 1:**
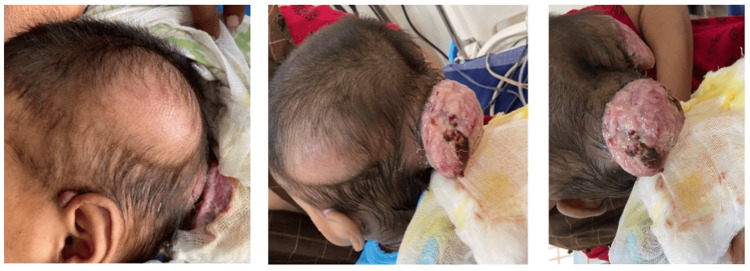
Clinical images showing three scalp swellings over the left parietal scalp and those over the right parietal and occipital areas with ulceration in an 18-month-old boy.

An 18-month-old boy presented to the department of dermatology at Belagavi Institute of Medical Sciences (India) with three scalp swellings that began as pea-sized swellings at 10 months of age. They progressively increased in size, ulcerated, and developed purulent discharge for five days. He was the fourth child of a couple with third-degree consanguineous marriage and was born via normal vaginal delivery following an uneventful full-term pregnancy. No family history of similar features was noted. Delayed physical developmental milestones and dentition were noted along with failure to thrive and difficulty in feeding. Mental development, anthropometric measurements, and vitals were normal. Auscultation revealed a 2/6 late systolic murmur in the pulmonic valve area. Laboratory tests revealed a platelet count of 820,000 cells/mm^3^ and anti-hepatitis C virus (HCV) antibodies. Three scalp swellings (5x6 cm; 6x7 cm; and 7x7 cm) were noted, which were soft-to-firm in consistency with central ulceration in two of them (Figure 1).

**Figure 2:**
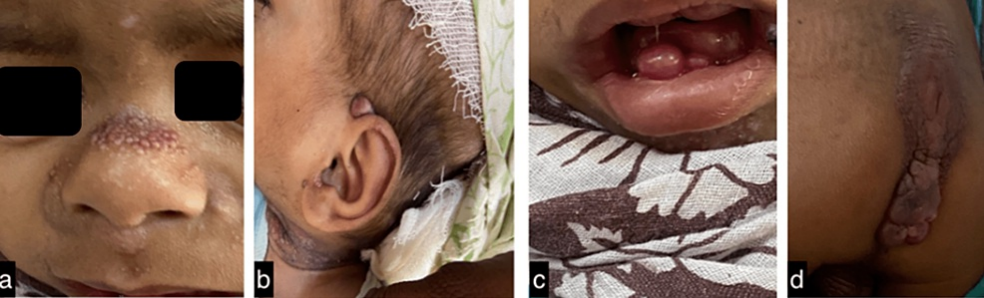
Clinical images in an 18-month-old boy of the pearly papules over the (a) nasal bridge and (b) neck, (c) Gingival hyperplasia, and (d) a 10x4-cm skin-coloured hypertrophic plaque over the lower back.

The swellings had non-fluctuating firm edges with a positive transillumination test. Other features included ulceration, discharge, and areas of granulation tissue and necrosis. Additionally, frontal bossing was noted with a depressed nasal bridge and multiple pearly pink papules over the bridge of the nose and the neck. An oral cavity examination revealed normal mucosa with gingival hyperplasia and delayed dentition. Furthermore, nodules were noted over both ears. Over the back, a hypertrophic verrucous skin-coloured plaque of 10 x 4 cm was noted extending from the lower back to the anal margin (Figures 2a-2d).

**Figure 3:**
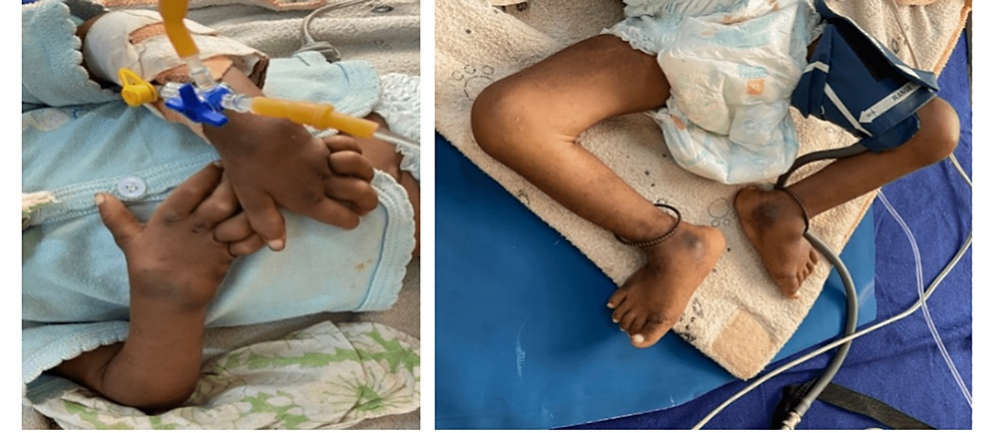
Clinical images showing musculoskeletal changes in an 18-month-old boy. Bilateral wrist deformity (left) with bilateral ankle valgus deformities (right).

On musculoskeletal examination, bilateral wrist contractures with subcutaneous nodules and bilateral valgus deformities at the ankle with hyperpigmentation over the malleoli were noted (Figure 3). Chest radiography was unremarkable.

**Figure 4:**
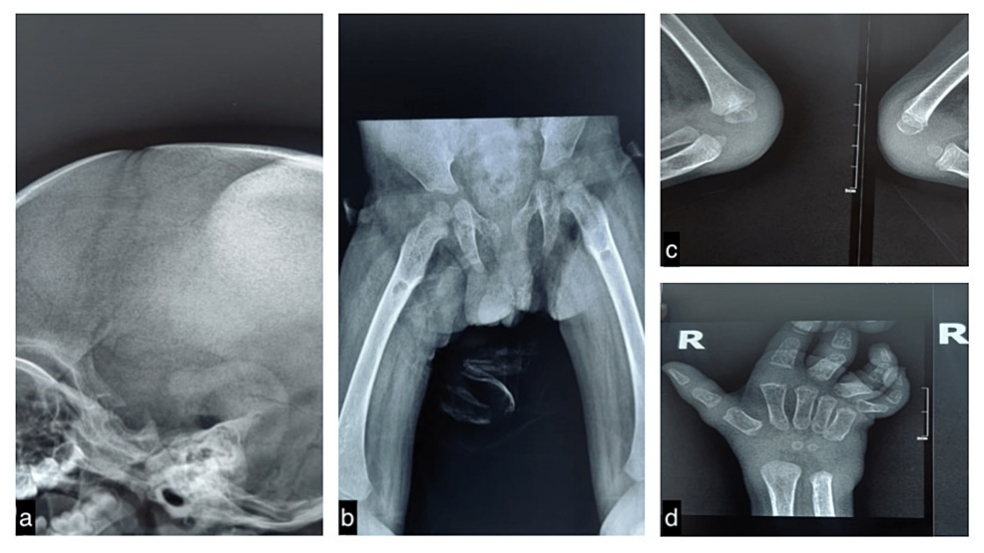
The skull radiograph shows a large soft tissue lesion in the parietal and occipital regions with no obvious signs of involvement of the underlying bone (a). Multiple symmetrical osteolytic areas in the proximal third of femoral shaft (b). Symmetrical osteolytic lesions with narrow zones of transition at the proximal tibial diaphysis and soft tissue opacities (c). Extension at the metacarpophalangeal joints, flexion at the interphalangeal joints, and soft tissue opacities around the wrist joints (d).

Skull radiography revealed a large soft tissue lesion in the occipito-parietal region with no underlying bone involvement (Figure 4a). Radiography of the hip joints revealed multiple symmetrical osteolytic areas in the proximal third of the femoral shaft (Figure 4b). Bilateral knee radiography (Figure 4c) revealed symmetrical osteolytic lesions with narrow zones of transition at the proximal tibial diaphysis and soft tissue opacities. Bilateral hand radiographs(Figure 4d) revealed extension at the metacarpophalangeal (MCP) joints and flexion at the interphalangeal joints (IPJs), flexion contractures of the third, fourth, and fifth MCP joints and IPJs. Additionally, soft tissue opacities were noted on the dorsal and ventral aspects of the wrist joints, which were suggestive of soft tissue nodules.

**Figure 5:**
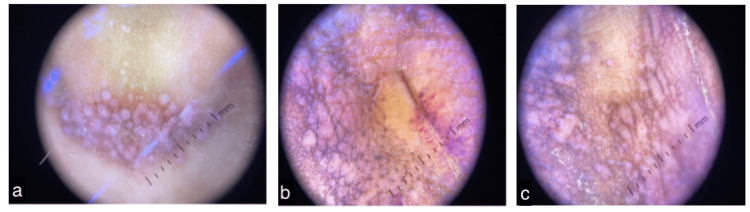
Dermoscopic images showing (a) pearly papules over the nose, (b) hypertrophic verrucous plaque over the lower back, and (c) pearly papules and plaques over the neck.

Dermoscopic examination of pearly papules over the nose, hypertrophic verrucous plaque over the back, and pearly papules and plaques over the neck (Figures 5a-5c) revealed pinkish-white globules with peripheral brown pigmentation. Few scattered grey dots were also seen along with a few reddish globules.

**Figure 6:**
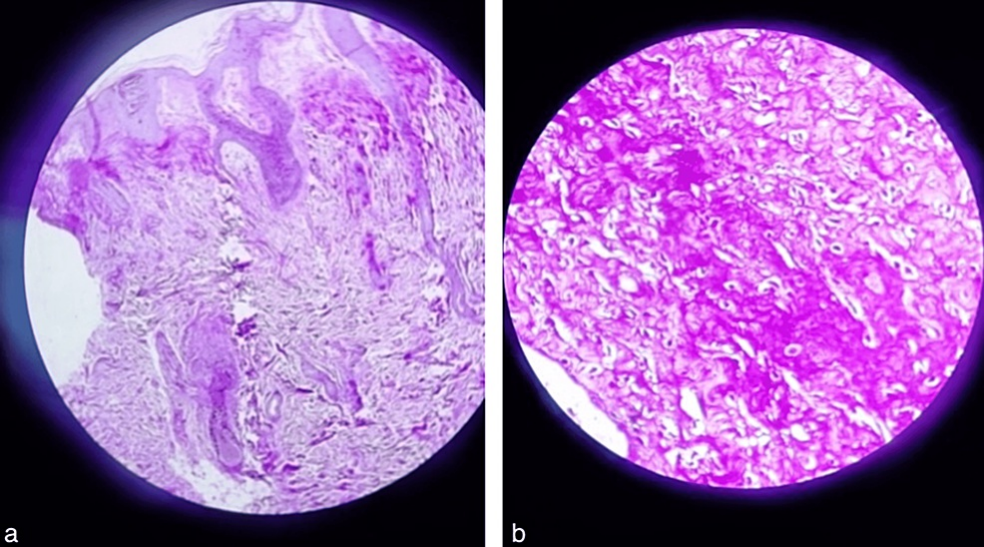
Histopathological images showing (a) periodic acid Schiff staining of the hyalinized stroma and (b) fibroblasts with bland nuclear features embedded in acellular eosinophilic hyalinized stroma.

Histopathological examination of the hypertrophic verrucous plaque revealed cartilage oligomeric matrix protein (COMP) fibroblasts with plump nuclei and bland nuclear features scattered in the abundant dense eosinophilic hyalinized stroma. Periodic Acid-Schiff (PAS) staining highlighted the hyalinized stroma. Excision biopsy of a scalp swelling revealed fibroblasts with bland nuclear features embedded in the acellular eosinophilic hyalinized stroma (Figures 6a, 6b). Additionally, few scattered vascular spaces and inflammatory cells were noted. PAS staining highlighted hyalinized stroma. These histopathological features were consistent with a diagnosis of JHF.

## Discussion

ISH and JHF are considered distinct clinical conditions [2-5]. In 2003, Dowling et al. and Hanks et al. established that ISH and JHF constitute a spectrum of the same disorder after identifying mutations in the capillary morphogenesis gene 2 (CMG2) on chromosome 4q21 [6]. Since they form a continuum with varying phenotypic expression, the encompassing term “hyaline fibromatosis syndrome” was proposed [1].

Joint contractures, papular and nodular skin lesions, gingival hypertrophy, osteopenia, and normal brain development are common clinical features of both ISH and JHF. Patients with ISH present within six months of life and typically die of infection or diarrhoea by two years of age, while patients with JHF present late in infancy or childhood with milder symptoms and usually survive till the second or third decade. Common distinguishing features of ISH include thickened skin, erythema or hyperpigmentation over bony prominences, visceral involvement, persistent diarrhoea, frequent severe infections, and failure to thrive. Patients with JHF, in contrast, tend to have larger nodules, commonly over the scalp [7,8]. Our patient had features of both ISH and JHF with an early onset of fleshy grouped papular lesions over the nose, ears, and gluteal region, and larger nodular lesions over the scalp with joint contractures and diarrhoea.

The pathogenesis of hyaline fibromatosis syndrome is unclear. It has been suggested that it may be due to an increased synthesis of glucosamine glycans by fibroblasts [3]. Deletion mutations in CMG2/anthrax toxin receptor 2 have been documented in patients with both JHF and ISH. CMG2 is an integrin‑like cell surface receptor that is believed to play a role in cell‑matrix interactions, basement membrane integrity, and endothelial cell morphogenesis [9]. Missense and in‑frame mutations that affect the cytoplasmic domain tend to result in JHF, whereas truncating mutations and missense mutations that affect the extracellular protein‑binding domain result in ISH [2]. While consanguinity is an important feature of history, it is not an invariable finding and does not interfere in diagnosing ISH and JHF in the right clinical settings.

Histologically, fibroblasts soaked in hyaline PAS+ material are noted. Cellularity is variable and inversely proportional to the intensity of hyaline deposits. Accumulation of hyaline material in the dermis is persistent and progressive, and lesions evolve from papules to nodules and tumors, which may ulcerate and become infected. Differential diagnoses of hyaline fibromatosis syndrome include congenital generalized fibromatosis, Farber lipogranulomatosis, lipoid proteinosis, mucopolysaccharidosis, and Winchester syndrome [10]. Symptomatic treatment is recommended for HFS. Non-steroidal anti-inflammatory drugs and opiates help with pain control. Physiotherapy should be included if passive movements of joint contractures are painful.

Most treatments in ISH have not proven to be beneficial. Surgical excision of large tumors is recommended but recurrences are common. Oral D‑penicillamine has been reported to improve joint mobility and flexibility. Gingival overgrowth may be treated with partial gingivectomy. Parenteral antibiotics and monitoring of fluid‑electrolyte balance may be required. Therapeutic trials with dimethyl sulfoxide, ketotifen, and calcitriol have been tried [5].

It is important to remember that the intellectual capacity in these patients is preserved. Therefore, early diagnosis, familial genetic counselling, and multi-disciplinary follow-up are required for improved outcomes. “Hyaline fibromatosis syndrome” is the best term that encompasses both ISH and JHF since no reliable distinction can be made between these conditions and overlapping features are common.

## Conclusions

Hyaline fibromatosis syndrome is a rare hereditary and progressive disease. It should be highly suspected in a patient with early onset papulonodular lesions, joint contractures, and gingival hypertrophy. Early diagnosis and proper multidisciplinary management are crucial in slowing the progression of this rare disabling disease.
